# Recent advances in the involvement of long non-coding RNAs in neural stem cell biology and brain pathophysiology

**DOI:** 10.3389/fphys.2014.00155

**Published:** 2014-04-22

**Authors:** Daphne Antoniou, Athanasios Stergiopoulos, Panagiotis K. Politis

**Affiliations:** Center for Basic Research, Biomedical Research Foundation of the Academy of AthensAthens, Greece

**Keywords:** non-coding genome, regulatory RNAs, gene regulatory networks, neural differentiation, neurogenesis, gliogenesis, brain-related diseases

## Abstract

Exploration of non-coding genome has recently uncovered a growing list of formerly unknown regulatory long non-coding RNAs (lncRNAs) with important functions in stem cell pluripotency, development and homeostasis of several tissues. Although thousands of lncRNAs are expressed in mammalian brain in a highly patterned manner, their roles in brain development have just begun to emerge. Recent data suggest key roles for these molecules in gene regulatory networks controlling neuronal and glial cell differentiation. Analysis of the genomic distribution of genes encoding for lncRNAs indicates a physical association of these regulatory RNAs with transcription factors (TFs) with well-established roles in neural differentiation, suggesting that lncRNAs and TFs may form coherent regulatory networks with important functions in neural stem cells (NSCs). Additionally, many studies show that lncRNAs are involved in the pathophysiology of brain-related diseases/disorders. Here we discuss these observations and investigate the links between lncRNAs, brain development and brain-related diseases. Understanding the functions of lncRNAs in NSCs and brain organogenesis could revolutionize the basic principles of developmental biology and neuroscience.

## Introduction

A long standing question in biological sciences is how cell diversity, specification patterns, and tissue complexity are generated during organ development. The mammalian brain is the most complex organ of any living organism. The question of how this enormous complexity is generated is still open. Such complexity is the result of millions of years of evolution that have equipped neural stem/progenitor cells (NSCs/NPCs) in the embryo with the ability to generate every neuron and glial cell in the brain via a combined action of extrinsic morphogenetic cues and intrinsic gene regulatory networks. For long time, it was thought that these networks are mainly based on cross-regulatory interactions between transcription factors (TFs) (Jessell, 2000; Politis et al., 2008; Kaltezioti et al., 2010; Martynoga et al., 2012; Stergiopoulos and Politis, 2013). However, the recent advent of sequencing methodologies and experimental data from large-scale consortia focused on characterizing functional genomic elements such as ENCODE and FANTOM, have revolutionized our view for organization, activity, and regulation of the mammalian genome (Carninci et al., 2005; Katayama et al., 2005; Birney et al., 2007). Surprisingly, the vast majority of the genome is transcribed producing not only protein-coding RNAs but also a vast number of different kinds of newly-identified classes of non-coding RNAs, including microRNAs (miRNAs-small non-coding RNAs) and long non-coding RNAs (lncRNAs). miRNAs have been extensively studied while the role of lncRNAs in cell biology together with novel roles of miRNAs in directly regulating lncRNAs have just begun to emerge (Amodio et al., 2012a,b; Rossi et al., 2013; Tay et al., 2014). LncRNAs are transcribed by RNA polymerase II, defined as endogenous cellular RNAs longer than 200 nt in length that lack an ORF, can be post-transcriptional processed by 5′ capping, polyadenylation, splicing, RNA editing, and exhibit specific sub-cellular localization (Qureshi and Mehler, 2012). Most importantly, it was recently shown that lncRNAs participate in the gene regulatory networks controlling embryonic stem cell (ESC) pluripotency and metastasis of cancer cells, as well as development and function of various tissues (Guttman et al., 2011; Gutschner and Diederichs, 2012; Qureshi and Mehler, 2012; Yang et al., 2013). Although lncRNAs are the most abundant classes of RNAs (Katayama et al., 2005; Birney et al., 2007; Kapranov et al., 2007b; Kapranov and St Laurent, 2012), they remain poorly characterized and their roles in brain development have just begun to arise. Notably, it has been proposed that the number of lncRNAs far exceeds the number of protein-coding mRNAs in the mammalian transcriptome (Carninci et al., 2005; Kapranov et al., 2010) and the vast majority of them appears to be expressed in adult brain in a highly specific manner (Qureshi and Mehler, 2012).

In the last few years, many groups have focused their efforts toward understanding the actions of lncRNAs in brain function and evolution. In this mini-review, we highlight the emerging evidence of the involvement of this new class of RNA molecules in NSC biology during development and adulthood, in health and disease. In particular, we summarize recent progress regarding the participation of lncRNAs in the regulatory networks that control the fine balance between proliferation and differentiation decisions of NSCs. The ability of lncRNAs to interact with both genomic loci and protein products of TF genes appears to endow them with a remarkable capacity to control NSC maintenance and differentiation, suggesting an undoubtedly important influence on the pathophysiology of several neurodegenerative disorders (Kapranov et al., 2007a,b; Guttman et al., 2009; Khalil et al., 2009; Orom et al., 2010; Bian and Sun, 2011; Qureshi and Mehler, 2012; Spadaro and Bredy, 2012; Ng et al., 2013).

## LncRNAs: new players in cell biology

LncRNAs can be classified as bidirectional, intronic, intergenic, sense, antisense or 3′-UTR (untranslated region) transcripts with respect to nearby protein-coding genes (Mercer et al., 2009; Derrien et al., 2012). Some lncRNAs are exported from nucleus and perform important functions in the cytoplasm, but more often they are found in nucleus, particularly associated with chromatin (Saxena and Carninci, 2011). In more detail, these transcripts may be bound by proteins, other RNA molecules or even fulfil intrinsic catalytic functions (Fedor and Williamson, 2005; Ye, 2007; Guttman and Rinn, 2012). The vast majority of these transcripts are not translated, supporting a role as non-coding RNAs rather than protein precursors (Banfai et al., 2012; Derrien et al., 2012). Furthermore, lncRNAs are developmentally regulated, expressed in specific cell types, associated with chromatin signatures indicating transcriptional regulation, are under evolutionary constraint and associated with carcinogenesis and other diseases; all of which support meaningful roles of lncRNAs (Qureshi and Mehler, 2012; Fatica and Bozzoni, 2014). LncRNAs also participate in a striking diversity of cellular processes including transcriptional regulation, genomic imprinting, alternative splicing, mRNA stability, translational control, DNA damage response, cell cycle regulation and organelle biogenesis (Rinn and Chang, 2012). Interestingly, it seems that knock-down or over-expression of many of these lncRNAs produce phenotypes that are very well correlated with their aberrant expression in disease states (Qureshi et al., 2010; Gutschner and Diederichs, 2012; Qureshi and Mehler, 2012). Therefore, we believe that lncRNA transcripts would eventually occupy a central place in our understanding of the molecular mechanisms underlying human diseases.

Systematical profiling and comprehensive annotation of lncRNAs in various cell types can shed light on the functionalities of these novel regulators. LncRNA-protein interactions can be currently predicted, approached and analyzed by various research advanced tools and strategies apart from bioinformatics, including high-throughput analysis of lncRNA expression [microarrays, RNA sequencing [RNA-Seq] (Mortazavi et al., 2008; Wang et al., 2009; Ilott and Ponting, 2013), RNA CaptureSeq, iSeeRNA [support vector machine (SVM)-based classifier] (Sun et al., 2013), BRIC-Seq [50-bromo-uridine (BrU) immunoprecipitation chase-deep sequencing analysis] (Imamachi et al., 2013)], RIP [RNA-binding protein (RBP) immunoprecipitation] coupled with microarray and high-throughput sequencing (RIP-Chip/Seq respectively) (Zhao et al., 2010; Jain et al., 2011), ChIRP (chromatin isolation by RNA purification) and CHART (capture hybridization analysis of RNA targets—High-throughput finding of RBPs and DNAs) as well as CLIP (crosslinking-immunopurification)-Seq, which provides transcriptome-wide coverage for mapping RBP-binding sites (Khalil et al., 2009; Wu et al., 2010; Chu et al., 2011; Zhu et al., 2013).

## LncRNAs in neural stem cells

These technologies have greatly helped the efforts to elucidate the role of lncRNAs in NSCs, neural differentiation, migration and maturation. Toward this goal, Ramos and colleagues identified and predicted regulatory roles for more than 12,000 novel lncRNAs (2,265 lncRNAs had proximal protein-coding gene neighbors) in the subventricular zone (SVZ) of adult mice. In FACS-sorted NSCs, they found a unique lncRNA expression pattern for each of the three stages of neurogenesis analyzed. Moreover, by using ChIP-Seq (chromatin immunoprecipitation-sequencing), lncRNAs were shown to be transcriptionally regulated in a manner analogous to mRNAs, enabling the prediction of lncRNAs that may function in the glial-neuronal lineage specification of multipotent adult NSCs (Ramos et al., 2013; Wang et al., 2013). Further transcriptome-next-generation-sequencing revealed that lncRNA-mediated alternative splicing of cell fate determinants controls stem-cell commitment during neurogenesis. Specifically, Aprea and co-workers identified several genic and intergenic lncRNAs that are functionally involved in neurogenic commitment, including *Cosl1*, *Btg2-AS1*, *Gm17566*, *Miat* (Myocardial infarction associated transcript-*Gomafu*), *Rmst* (Rhabdomyosarcoma 2 associated transcript), *Gm17566*, *Gm14207*, *Gm16758*, *2610307P16Rik*, *AC102815.1*, *C230034O21Rik* and *9930014A18Rik* (Aprea et al., 2013). Accordingly, subsequent studies using high-throughput transcriptomic data (microarray platform) to examine lncRNA differential expression in NSCs, GABAergic neurons and oligodendrocytes, led to the identification of lncRNAs that are dynamically regulated during neural lineage specification, neuronal-glia fate switching and oligodendrocyte maturation [i.e., *Dlx1AS* (Distal-less homeobox 1 antisense), *Evf2* (Embryonic ventral forebrain 2), *Rmst*, *utNgn1* (untranslated Neurogenin1), *MALAT1* (metastasis-associated lung adenocarcinoma transcript 1; also named *NEAT2*)] (Mercer et al., 2010; Qureshi and Mehler, 2012; Ng et al., 2013). ChIP-Seq approaches were also developed to generate and investigate genome-wide chromatin-state maps. These functional analyses predicted hypothetical roles for 150 mammalian lincRNAs (long intergenic non-coding RNAs) in a “guilt-by-association” manner in NSCs. In particular, specific lincRNAs appeared to localize near genes encoding key TFs (such as *Sox2*, *Klf4*, *Myc*, *p53*, *NFkB* and *Brn1*) that are involved in processes ranging from hippocampal development to neuronal and oligodendrocyte maturation (Guttman et al., 2009; Qureshi and Mehler, 2012).

**Figure 1:**
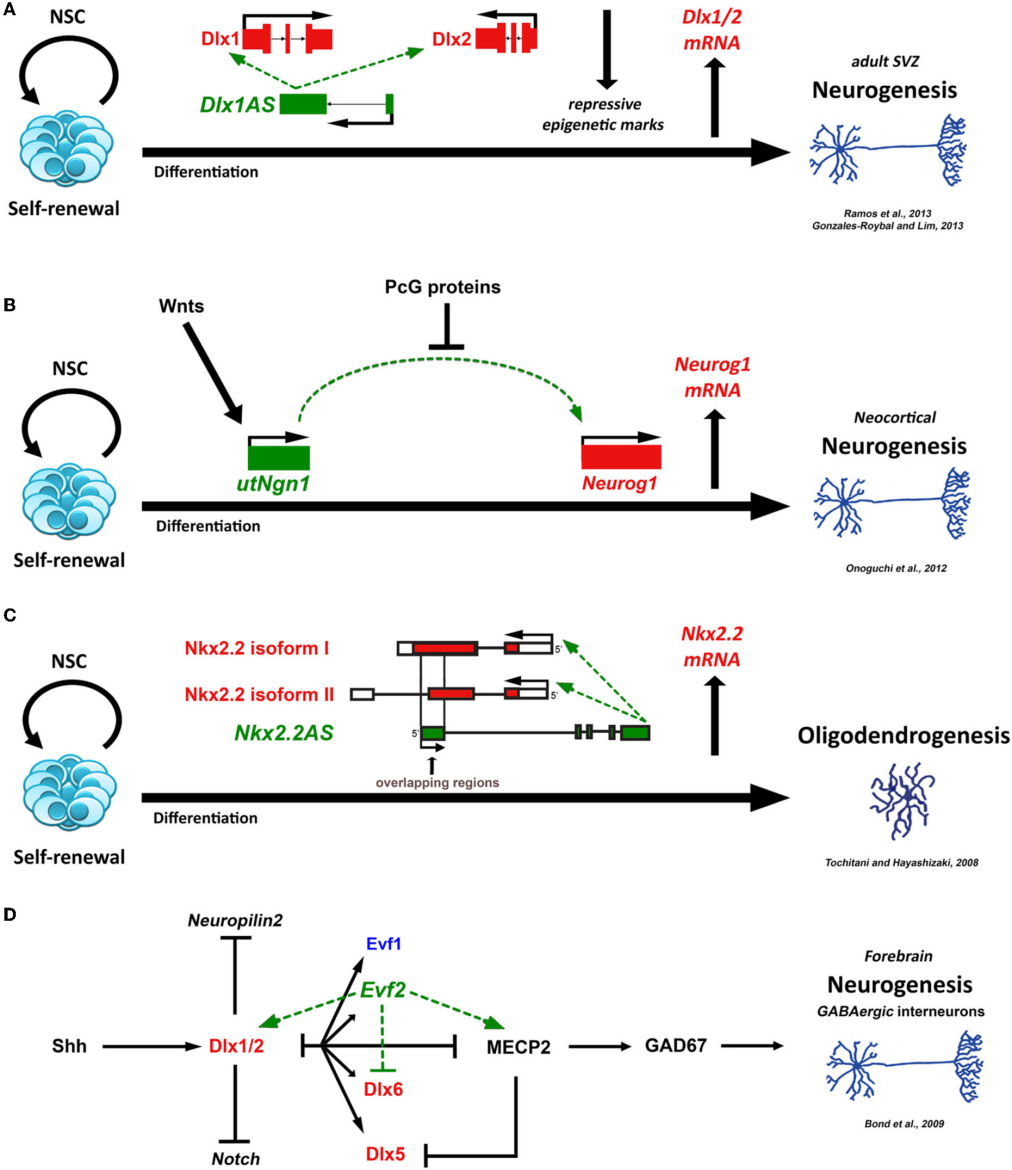
**Proposed schematic models for the role of different lncRNAs (green) in neural development. (A)** LncRNA *Dlx1AS* consists of two exons that are spliced and the mature transcript is polyadenylated. During neuronal differentiation of adult SVZ NSCs, *Dlx1AS* is required for the induction of *Dlx1* and *Dlx2* (red) gene expression (Gonzales-Roybal and Lim, 2013; Ramos et al., 2013). **(B)** During neocortical neurogenesis, *utNgn1* receive Wnt signals (i.e., Wnt3a) to induce the expression of *Neurog1* (red). PcG protein-mediated mechanisms (i.e., Ring1B, H3K27me3, H3K4me3, H3K9/K14ac) lead to the suppression of *utNgn1* (Onoguchi et al., 2012). **(C)** The overlapping regions of *Nkx2.2AS* and *Nkx2.2* isoforms (*I* and *II*-red) are necessary to promote *Nkx2.2* mRNA levels and the subsequent oligodendrocytic differentiation of NSCs (Tochitani and Hayashizaki, 2008). **(D)** Mechanistic pathway for *Evf2*-dependent interactions crucial for forebrain development. Secreted Shh (Sonic hedgehog) promotes expression of *Dlx1* and *Dlx2* (red), which sequentially suppress *Neuropilin2* and Notch signaling. Activation of *Evf2* leads to the formation of a regulatory network together with *Dlx*'*s* (red) and *MECP2* (methyl CpG binding protein 2) that controls *GAD67* and GABAergic-interneuron formation (Bond et al., 2009). NSC, neural stem cell; *Evf1* (lncRNA) (blue), Embryonic ventral forebrain-1, Dlx6 antisense RNA 1 (*Dlx6AS1*).

More concisely, Ramos et al. have also performed *in vitro* knock-down studies and observed that *Dlx1AS*, a lncRNA encoded from a bigene *Dlx1/2* cluster, is associated with fate determination of adult SVZ NSCs via positive regulation of *Dlx1* and *Dlx2* gene expression. Mechanistically, enhanced transcription of *Dlx1AS* occurs during neurogenesis when H3K27me3 (trimethylation of histone H3 Lys-27) repression is decreased. The H3K27me3-specific demethylase JMJD3 was also found to be enriched at the *Dlx1AS* locus (Figure 1A) (Gonzales-Roybal and Lim, 2013; Ramos et al., 2013). Recently, a comprehensive study came up with the finding that *UtNgn1* is a non-coding RNA transcribed from an enhancer region of the *Neurogenin1* (*Neurog1*) locus. This non-coding transcript seems to positively regulate *Neurog1* transcription during neuronal differentiation of NSCs. In detail, during the late stage of neocortical NSC development, *utNgn1* expression is up-regulated via involvement of Wnt signaling whereas it is down-regulated by PcG (polycomb group) proteins (Figure 1B) (Onoguchi et al., 2012). Additionally, two lncRNAs, nuclear enriched abundant transcripts *NEAT1* and *NEAT2* (*MALAT1*) are up-regulated in neuronal and glial progeny, and are associated with neuronal activity, growth and branching (Mercer et al., 2010; Ng et al., 2013). *Miat* (also called *Gomafu*) is one of the known non-coding RNAs that participates in neuronal subtype- specific determination and is also highly expressed in oligodendrocyte lineage specification (Aprea et al., 2013). In the same lineage, another lncRNA, *Nkx2.2AS*, an antisense RNA of the known homeobox TF *Nkx2.2*, appears to lead NSCs into oligodendrocytic fate through modest induction of *Nkx2.2* gene. By using deletion mutants, Tochitani and Hayashizaki showed that the overlapping regions of *Nkx2.2AS* and *Nkx2.2* isoforms are required for promoting *Nkx2.2* mRNA levels and the subsequent oligodendrocytic differentiation of NSCs (Figure 1C) (Tochitani and Hayashizaki, 2008). *Evf2* is predominantly detected in the developing forebrain, both in human and mouse, and is critical for GABAergic-interneuron formation. Like other lncRNAs, *Evf2* seems to control the expression of specific genes that are necessary during brain development, such as *Dlx5*, *Dlx6* and *Gad1*, through distinct *trans*- and *cis*-acting mechanisms (Figure 1D) (Bond et al., 2009). Interestingly, *Rmst* is presented to be a novel marker for dopaminergic neurons during NSC differentiation, where it is co-expressed with the midbrain-specific TF *Lmx1a* (Uhde et al., 2010).

Considering the tight connection between pluripotency of human ESCs and their differentiation into NSCs (ESC-derived NSCs) and subsequently into neurons, Ng and co-workers extensively showed that diverse lncRNAs are essential for ESC proliferation and neurogenesis through physical interaction with master TFs (e.g., *Sox2*). In detail, microarray data analysis led to the identification of 35 neuronal lncRNAs with important roles in neuronal differentiation, e.g., *Rmst* (*AK056164*, *AF429305* and *AF429306*), *lncRNA_N1* (*AK124684*), *lncRNA_N2* (*AK091713*), *lncRNA_N3* (*AK055040*, etc.). They also suggested that these lncRNAs are required for generation of neurons and suppression of gliogenesis by association with chromatin modifiers and nuclear proteins (Ng et al., 2012). In agreement, another study that aimed at identifying functional features of lncRNAs during ESC differentiation to adult cerebellum by utilizing RNA-Seq and ChIP-Seq technologies, demonstrated that filtered novel lncRNAs are located close to protein-coding genes involved in functions such as neuronal differentiation and transcriptional regulation (Guttman et al., 2009; Lv et al., 2013). Particular studies have been successful in identifying *anti-NOS2A* as an antisense transcript of the *NOS2A* (Nitric oxide synthase 2 enzyme) gene, an isoform of the NOS protein which induces hESC differentiation into neurogenic precursors (Korneev et al., 2008). Specific expression pattern was also detected for *Otx2c* (Orthodenticle homeobox 2 c), an alternative splicing variant of the pre-mRNA *Otx2* with a possible role in neural differentiation of hESCs (Liu et al., 2013). Another lncRNA that is correlated with the proliferation state of ESCs is *lincRNA-Sox2*, which is located at the promoter of *Sox2* locus and is regulated by *Sox2* and *Oct4* (Guttman et al., 2009). Finally, a thorough targeted RNA-Seq analysis carried out using neurons derived from patient-specific induced pluripotent stem cells (iPSCs) showed that more than 1,500 lncRNAs are dynamically regulated during differentiation of iPSCs toward glutamatergic neurons. Particularly, the expression of 1,622 non-coding genes (lncRNAs/lincRNAs) was dramatically affected during conversion from iPSCs to differentiating neurons, while alternative splicing occurred. Significant alterations were also observed in the expression patterns of non-coding genes involved in neuropsychiatric disorders (Lin et al., 2011; Qureshi and Mehler, 2012; Akula et al., 2014).

## LncRNAs in brain function, evolution and neurological diseases

The importance of lncRNAs in the brain is explicitly highlighted by the observation that most of them are expressed in the adult mammalian brain in highly regional-, cellular- and sub-cellular compartment-specific and neuronal activity dependent profiles (Mercer et al., 2008; Ponjavic et al., 2009; Qureshi and Mehler, 2012). In fact, it seems that lncRNAs have played important roles in the evolution of the form and function of human brain, since the fastest evolving regions of the primate genome are non-coding sequences that generate lncRNAs implicated in the modulation of neuro-developmental genes (Pollard et al., 2006; Qureshi and Mehler, 2012). The most dramatic of these regions, called *HAR1* (human accelerated region 1), is part of a novel lncRNA gene (*HAR1F*) that is expressed specifically in Cajal–Retzius neurons in the developing human neocortex from 7 to 19 gestational weeks, a crucial period for cortical neuron-specification and migration. Additionally, analysis of highly conserved lncRNAs in birds, marsupials and eutherian mammals revealed a remarkable similarity in spatiotemporal expression profiles of orthologous lncRNAs, suggesting ancient roles during brain development (Chodroff et al., 2010). Moreover, lncRNAs directly modulate synaptic processes (*MALAT1*), protein synthesis (*BC1*), learning and memory (*Tsx*), and are associated with neuronal activity in mouse cortex (Qureshi and Mehler, 2012). In agreement with these effects on neurons, lncRNAs have also been implicated in the pathophysiology of neuro-developmental, neurodegenerative, neuro-immunological and neuro-oncological diseases/disorders. For instance, high levels of *BC1*/*BC200* and *BACE1-AS* have been implicated in Alzheimer's disease (AD) while *NEAT1*'s in Huntington's disease (HD). *BC1*/*BC200* normally seems to selectively regulate local protein synthesis in post-synaptic dendritic compartments, by repressing translation via an *elF4*-*A*-dependent mechanism (Qureshi et al., 2010; Niland et al., 2012). De-regulation of *BACE1-AS* has been shown to be responsible for feed-forward induction of BACE1 (β-secretase); thus leading to amyloid β (Aβ) increased production and possible AD pathogenesis (Qureshi et al., 2010). Additionally, *NEAT1* is a lncRNA known to be involved in cell death mechanisms. In particular, *NEAT1* controls target gene transcription by protein sequestration into paraspeckles (stress-responsive sub-nuclear structures), a potentially dysfunctional pathway in HD progression. In accordance, Hirose and co-workers have very recently shown that *NEAT1* transcriptional up-regulation leads to the enlargement of these structures after proteasome inhibition (Johnson, 2012; Hirose et al., 2014). Various lncRNAs have also been correlated with Parkinson's disease (*PINK-AS1*, *UCHL1-AS1*), amyotrophic lateral sclerosis (*MSUR1*), Down's syndrome (*NRON*) as well as with tumor progression (*ANRIL*, *MALAT1*, *HOTAIR*, *NOS3AS*) (Gutschner and Diederichs, 2012). A common theme emerging from all these cases is that lncRNAs control the expression of nearby protein-coding genes *in cis* and de-regulation of this relationship could lead to nervous system-related diseases. In agreement, brain-expressed lncRNAs are preferentially located adjacent to protein-coding genes that are also expressed in the brain and involved in transcriptional regulation and/or nervous system development (Mercer et al., 2008; Ponjavic et al., 2009). Many of these pairs often exhibited coordinated expression during developmental transitions. Therefore, it was suggested that these lncRNAs may influence the expression of the associated protein-coding genes similarly to previously characterized examples such as *Nkx2.2AS*, *HOTAIR*, *p15AS*, *p21AS* and *Evf2*.

## Perspective links between lncRNAs and brain development

Over recent years, it has become more and more evident that lncRNAs play key roles in gene regulatory networks controlling nervous system development. To initially investigate this link, we bioinformatically screened numerous mouse genes encoding TFs with well-established roles in CNS (central nervous system) development for close proximity with lncRNA genes (based on publicly available data from UCSC genome-browser). Accordingly, we managed to identify 79 such TF genes encompassing 91 transcriptional units for lncRNA genes in close proximity (<3,000 base pairs-bp) (Table 1). In particular, 40.65% of these lncRNAs are bidirectional, 16.48% intronic, 9.89% intergenic, 12.08% sense, 15.38% antisense and 5.49% 3′-UTR. The majority of these lncRNAs have only been identified in genome-wide expression screens, are expressed in nervous system, and intriguingly, their functions are totally unknown. Some of them have been reported to interact with TFs or chromatin-associated complexes (Wang and Chang, 2011; Guttman and Rinn, 2012; Rinn and Chang, 2012), indicating potential contribution to the combinatorial transcriptional codes involved in NSC maintenance, sub-type/lineage specification and terminal differentiation. Based on these preliminary evidence, we postulate that lncRNAs and TFs with regulatory role in neurogenesis are inter-connected and may form coherent cross-regulatory networks that are associated with CNS development. However, this hypothesis needs to be extensively interrogated in NSCs. Moreover, considering the fact that some TFs are used as reprogramming tools for the production of iPSCs, induced neurons, induced NSCs, cardiomyocytes, etc. (Yang et al., 2011), these lncRNAs may be utilized for the production of clinically useful cell types in the near feature, revolutionizing the basic principles of developmental/cell biology as well as neuroscience.

**Table 1:** **List of genes encoding TFs with critical roles in brain development that also contain lncRNA genes in close proximity to their genomic loci (<3,000 bp)**.

**Genes encoding for TFs**	**LncRNAs**	**Position**
Ascl4	AK018959	*Sense*
Atoh7	AK005214	*Sense*
Crebbp	4930455F16Rik	*Intergenic*
Crx	CrxOS	*Bidirectional*
Ctnnb1	4930593C16Rik	*Bidirectional*
Cux2	AK006762	*Sense*
Cux2	AK187608	*Bidirectional*
Dlx1	Dlx1AS	*Antisense*
Dlx4	A730090H04Rik	*Bidirectional*
Dlx6	Dlx6AS1	*Antisense*
Dlx6	Dlx6AS2	*Intronic*
Emx2	Emx2OS	*Bidirectional*
Evx1	5730457N03Rik	*Bidirectional*
FezF1	FezF1-AS1	*Bidirectional*
FezF1	AK086573	*Intergenic*
FoxA2	AK156045	*Antisense*
FoxG1	AK158887	*Intergenic*
FoxG1	3110039M20Rik	*Sense*
Gata1	S57880	*Sense*
Gata2	AK137172	*Sense*
Gata3	4930412013R	*Bidirectional*
Gata4	AK031341	*Intronic*
Gata6	AK033147	*Bidirectional*
Gata6	AK003136	*Bidirectional*
Gbx2	D130058E05Rik	*Bidirectional*
Gli1	AK157048	*Sense*
Gli2	AK054469	*Intronic*
Gli3	AK135998	*Intergenic*
Hmx1	E130018O15Rik	*Bidirectional*
Irx2	Gm20554	*Bidirectional*
Lef1	Lef1-AS1	*Bidirectional*
Lhx1	Lhx1OS	*Bidirectional*
Lhx3	AK035055	*Bidirectional*
Lhx8	AI606473	*Bidirectional*
Lmx1b	C130021I20	*Bidirectional*
Lxrb (NR1H2)	AK184603	*3 ′-UTR*
Meis1	AK144295	*Antisense*
Meis2	AK012325	*Sense*
Meis2	AK144367	*Bidirectional*
Meis2	AK144485	*Bidirectional*
Msx1	Msx1AS	*Antisense*
MycN	MYCNOS	*Bidirectional*
Myt1L	AK138505	*Antisense*
NFATc1	AK155068	*3 ′-UTR*
NFATc4	AK014164	*Sense*
NFIA	E130114P18Rik	*Bidirectional*
NFIB	AK081607	*Intronic*
NFIx	AK168184	*Antisense*
NFkB2	AK029443	*Bidirectional*
Ngn1	AK016084	*Intergenic*
Nkx2.2	Nkx2.2AS	*Antisense*
Notch1	AK075572	*Antisense*
NR2F1	AK051417	*Intergenic*
NR2F1	A830082K12Rik	*Bidirectional*
NR2F2	AK135306	*Intronic*
NR3C2	Gm10649	*Bidirectional*
NR4A2	BB557941	*Intergenic*
NR5A2 (lrh-1)	AK178198	*Antisense*
NR5A2 (lrh-1)	AK145521	*Intronic*
Otx2	Otx2OS	*Bidirectional*
Pax2	AK006641	*Antisense*
Pax6	AK044354	*Antisense*
Pbx3	AK138624	*Intronic*
Pou4F1	AK084042	*Intergenic*
PPARd	AK033897	*Intronic*
PPARd	AK007468	*Intronic*
Prox1	AK142161	*Bidirectional*
Ptf1a	AK053418	*Antisense*
RARa	AK031732	*Intronic*
RARb	AK052306	*3 ′-UTR*
RBPjK	AK164362	*Intronic*
Runx1	AK131747	*Intergenic*
SATB2	9130024F11Rik	*Bidirectional*
Six1	AK035085	*3 ′-UTR*
Six3	Six3OS1	*Bidirectional*
Six6	4930447C04Rik	*Bidirectional*
Sox1	Gm5607	*Sense*
Sox10	GM10863	*Bidirectional*
Sox2	Sox2OT	*Sense*
Sox21	AK039417	*Bidirectional*
Sox8	AK079380	*Bidirectional*
Sox9	BC006965	*Bidirectional*
STAT5b	AK088966	*Intronic*
Tgif2	5430405H02Rik	*3 ′-UTR*
THRA	AK165172	*Intronic*
THRB	AK088911	*Antisense*
WT1	AK033304	*Intronic*
WT1	AI314831	*Bidirectional*
Zeb1	AK041408	*Intronic*
Zeb1	Gm10125	*Bidirectional*
Zeb2	Zeb2OS	*Bidirectional*

Some of these TFs contain more than one lncRNA near to their genes (duplicates). The position of each lncRNA relatively to its nearby protein-coding gene is indicated at the third column of the table.

**Figure 2:**
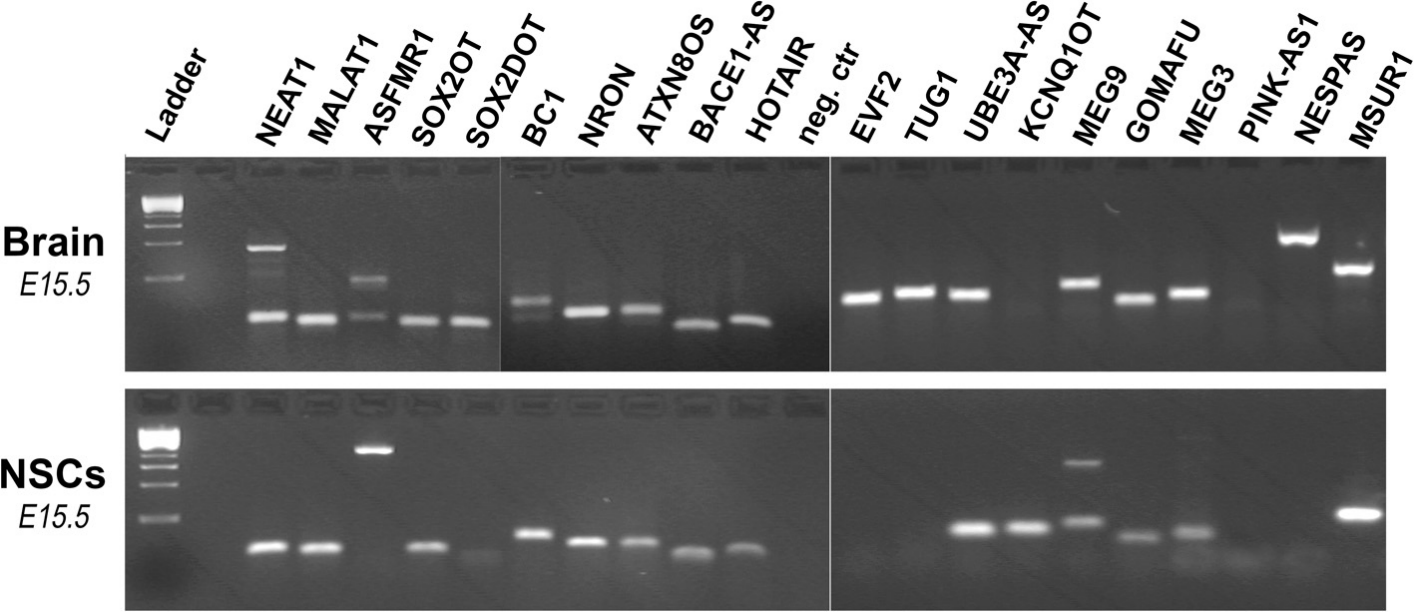
**RT-PCR-based detection of various lncRNAs associated with CNS-related diseases or disorders**. Experiments were performed using cDNA samples derived from embryonic mouse brain (*E15.5*) or NSCs isolated from embryonic mouse telencephalon and cultured *ex vivo*, as indicated. In some cases alternative splicing isoforms are also evident. RT-PCR primer sequences are available upon request.

To further examine the links between lncRNAs, brain development and neurological disorders, we tested whether lncRNAs, associated with nervous system-related diseases in humans, are expressed in embryonic mouse brain (*E15.5*) or *ex vivo* cultured NSCs (isolated from mouse telencephalon, *E15.5*). The rational for these experiments was based on observations showing many neurological diseases to be initiated very early during brain maturation and development (Bian and Sun, 2011; Qureshi and Mehler, 2012). Specifically, we first identified *in silico* and then tested for expression 20 such lncRNAs with clear conservation in mouse genome. Interestingly, RT-PCR (reverse transcription-PCR) analysis revealed that most lncRNAs are expressed in both brain and NSCs (Figure 2), suggesting potential involvement in brain development. Moreover, a possible function of these lncRNAs in NSCs may contribute to their roles in neurological diseases. Nevertheless, further investigation and initiation of new experimental studies are needed to uncover the exact function of lncRNAs in NSCs and nervous system in general.

## Conclusions

LncRNAs represent a new exciting frontier in molecular biology with major roles in NSC fate decisions, specification and commitment. Full understanding of how these non-coding transcripts regulate the expression of protein-coding genes and participate in gene regulatory circuitries could lead to the discovery of novel cellular/molecular mechanisms and signaling pathways involved in neural development; thus having potential implications for the treatment of nervous system-related diseases and traumas.

### Conflict of interest statement

The authors declare that the research was conducted in the absence of any commercial or financial relationships that could be construed as a potential conflict of interest.

## Abbreviations

lncRNA: long non-coding RNA
lincRNA: large intergenic non-coding RNA
miRNA: microRNA
NSCs: neural stem cells
ESCs: embryonic stem cells
iPSCs: induced pluripotent stem cells
CNS: central nervous system
SVZ: subventricular zone
ChIP: chromatin immunoprecipitation
RBP: RNA binding protein
RNA-Seq: RNA sequencing
RIP-Chip: RBP immunoprecipitation-microarray (Chip)
ChIRP: chromatin isolation by RNA precipitation
CHART: capture hybridization analysis of RNA targets
CLIP: crosslinking-immunopurification
UCSC: University of California Santa Cruz
ORFs: open reading frames
TFs: transcription factors
HAR1: human accelerated region 1
AD: Alzheimer's disease
HD: Huntington's disease
*Dlx1AS*: distal-less homeobox 1 antisense
*Miat*: myocardial infarction associated transcript
*Rmst*: rhabdomyosarcoma 2 associated transcript
*Evf2*: embryonic ventral forebrain 2
*UtNgn1*: untranslated neurogenin1
*NEAT*: non-coding nuclear enriched abundant transcript.
